# Evaluation of Anti-Cytotoxic and Anti-Genotoxic Effects of *Nigella sativa* through a Micronucleus Test in BALB/c Mice

**DOI:** 10.3390/nu12051317

**Published:** 2020-05-06

**Authors:** Raúl S. Franco-Ramos, Carlos A. López-Romero, Hugo Torres-Ortega, Darío Oseguera-Herrera, Jose P. Lamoreaux-Aguayo, Daniel Molina-Noyola, Clara I. Juárez-Vázquez, Olivia Torres-Bugarín

**Affiliations:** 1 Facultad de Medicina, Universidad Autónoma de Guadalajara, Zapopan 45129, JAL, Mexico; francoraul57@gmail.com (R.S.F.-R.); carloslopezrom_98@hotmail.com (C.A.L.-R.); htoucsc@gmail.com (H.T.-O.); darioseguerah@hotmail.com (D.O.-H.); josepablolamoreaux99@gmail.com (J.P.L.-A.); 2 Laboratorio de Evaluación de Genotóxicos, Programa Internacional de Medicina, Universidad Autónoma de Guadalajara, Zapopan 45129, JAL, Mexico; daniel.mn1234@gmail.com; 3 Facultad de Medicina, Decanato Ciencias de la Salud, Universidad Autónoma de Guadalajara, Zapopan 45129, JAL, Mexico; clara.juarez.vazquez@gmail.com

**Keywords:** *Nigella sativa*, thymoquinone, cisplatin, micronucleus test, micronuclei, Howell–Jolly bodies, anti-cytotoxicity, anti-genotoxicity, BALB/c mice, bone marrow

## Abstract

*Nigella sativa* (*N. sativa*) is a medicinal plant used for its therapeutic pharmacological effects such as anti-inflammatory, antioxidant, anticancer, antidiabetic, and immunomodulation. This study explored the anti-cytotoxic and anti-genotoxic effect of *N. sativa* through a micronucleus test (MNT) of BALB/c mice peripheral blood. Using 6-to-8-week-old healthy male BALB/c mice, four groups were formed: (1) Control (sterile water), single-dose 2 mg/kg/intraperitoneal (i.p); (2) *N. sativa* oil, 500 mg/kg/24 h/7 days/i.p; (3) Cisplatin (CP), single-dose 2 mg/kg/subcutaneous (s.c); (4) *N. sativa* + CP with their respective dosage. When evaluating polychromatic erythrocytes (PCE), a biomarker of cytotoxicity, the group treated with *N. sativa* + CP experienced an increase in the frequency of PCE, which demonstrated the recovery of bone marrow and modulation of cell proliferation. The analysis of micronucleated polychromatic erythrocytes (MNPCE), an acute genotoxicity biomarker, showed similar frequency of MNPCE within the groups except in CP, but, in the *N. sativa* + CP group, the frequency of MNPCE decreased and then regulated. Finally, the frequency of micronucleated erythrocytes (MNE), a biomarker of genotoxicity, the supplementation of *N. sativa* oil did not induce genotoxic damage in this model. Thus, we conclude that *N. sativa* has both cytoprotective, genoprotective effects and modulates cell proliferation in BALB/c mice.

## 1. Introduction

For many centuries, medicinal plants have been used in the treatment of numerous diseases and illnesses. *Nigella sativa (N. sativa)*, also known as black cumin, is native to Southern Europe, North Africa, and Southeast Asia [1,2,3,4], and, for its therapeutic pharmacological effects, it is cultivated in many countries around the world. To this day, many research studies have been carried out on *N. sativa* to better comprehend its many medicinal properties and to investigate its potential use as an adjuvant in pharmacological treatments. *N. sativa* seeds are rich in proteins (26.7%), fat (28.5%), carbohydrates (24.9%), crude fiber (8.4%), total ash (4.8%), vitamins, and minerals (Cu, P, Zn & Fe) [1,2,4,5]. The chemical compounds isolated from *N. sativa* seeds are quinine, carvone, citronellol, and limonene [1,2,3,4,6,7,8]. Most of the pharmacological properties of *N. sativa* are attributed to quinine constituents, of which thymoquinone is the most abundant (30–40%) [1,2,3,4,5,6,7,8]. From the numerous studies done on *N. sativa*, the reported therapeutic pharmacological effects are diuretic, analgesic, anthelmintic, bronchodilator, spasmolytic, gastroprotective, hepatoprotective, as well as anticancer, antioxidant, antihypertensive, antidiabetic, and immunomodulation [1,2,3,4,6,9,10,11,12,13,14].

The biological and pharmacological effects of *N. sativa* have been well established, and the present study explored the anti-cytotoxic and anti-genotoxic effect of *N. sativa* through micronuclei testing of BALB/c mice peripheral blood. In the present study, the micronucleus test (MNT) was used because, for many years, it has been used to evaluate the loss of genetic material in a clear, accurate, fast, and cost-effective way [15,16,17,18]. It has been used and is widely accepted for in vitro and in vivo genotoxicity research as it serves as a sensitive marker of genomic damage [16,17,18]. Micronuclei (MN) also known as Howell–Jolly bodies, are formed during the transition metaphase-anaphase of mitosis because of chromosome breakage or malformation of the mitotic spindle, which is caused by aneugenic and/or clastogenic mechanisms [16,17,18,19,20,21]. The etiology of these events can be explained by the production of free radicals during an oxidative stress process. The oxidative stress process can be seen in chronic diseases such as diabetes, rheumatoid arthritis, cardiovascular diseases, Alzheimer’s, and cancer. Moreover, in the presence of certain endogenous [15,22,23] or exogenous [14,22,23,24,25] factors, MN increases in frequency, which is a sign of genotoxic events and chromosomal instability [26,27,28]. Thus, micronucleated erythrocytes (MNE) can be used as a biomarker to track through cell morphology and deduce the mutagenic influence the genotoxic agents have on an organism [29,30,31]. Furthermore, it can be used to evaluate the beneficial effects of adjuvants in the treatment of chronic diseases [22]. The MNT is performed through the visualization of cell morphology with a microscope after the smear is fixed and stained [21,32].

The primary function of bone marrow is to produce erythrocytes, platelets, and leukocytes. Based on the needs of the body, the amount and rate of production of hematopoietic cells may vary [33]. Reticulocytes are immature erythrocytes; therefore, the reticulocyte count is used to monitor the process of erythropoiesis [33,34]. More specifically, the reticulocyte count is used to evaluate bone marrow function in response to internal or external stimuli [34,35]. In MNT, reticulocytes are called polychromatic erythrocytes (PCE) because they contain RNA and can be differentiated from total erythrocytes with staining techniques [34]. The frequency of PCE reflects the activity of bone marrow [34,35,36]. Reticulocytes develop and mature in the bone marrow and then circulate for about 24 h in the bloodstream before developing into mature erythrocytes, which occurs in the spleen [33,34,35,36]. An additional parameter to consider is the formation of micronucleated polychromatic erythrocytes (MNPCE), which occurs when the nucleus is pushed out, but chromosomal material remains in the cytoplasm indicating acute damage to bone marrow. 

Because the MNT serves as a sensitive marker of genomic damage [22], in the present study, it was used to evaluate the toxicity as well as the anti-cytotoxic and anti-genotoxic effects of *N. sativa* in comparison to a widely used drug that is already known to cause toxicity in the organism. Such a drug is Cisplatin (CP), an alkylating agent used in the treatment of various forms of cancer and solid tumors [23,37,38]. However, the induced generalized toxicity caused by CP in the organism has been a major setback in its use [38]. The toxic effects of CP on the organism are seen after a single dose in approximately 28% to 36% of cancer patients [23]. Regarding the mechanism of action of CP, its small molecular structure allows it to easily cross the plasma membrane and then to the nucleus. Once in the nucleus, it creates inter-strands and intra-strands cross-links in cellular DNA which consequently derail the replication fork and transcription machinery leading to the killing of the affected cells [23,37,38]. 

Many research studies suggest that a combination of therapy with *N. sativa* and its active quinine constituents, of which thymoquinone is the most abundant, prevent weakening of the immune system and minimize oxidative damage and other toxic side-effects of cytotoxic drugs on healthy cells, thus permitting their recovery [39]. In addition, it has been reported that *N. sativa* and its active constituents cause cell cycle arrest, suppressing the progression from G1 to S phase by targeting cyclin E, cyclin D1, and p27 proteins [39]. Furthermore, other studies explain other mechanisms of *N. sativa*, which can cause apoptosis in malignant cells by producing and accelerating the reactive oxygen species (ROS), DNA cleavage, immunomodulation, telomeric attrition, autophagy induction, and rectify the signaling pathways [39]. More specifically the antioxidant mechanism of thymoquinone, the most abundant constituent of *N. sativa,* has been reported to act as a cytoprotective agent against alkylating agents via maintaining hemoglobin concentrations, normalizing sugar concentrations, lowering alkaline phosphatase, kidney parameters such as creatinine, urea, bilirubin, cholesterol, low-density triglycerides as well as lipid per-oxidation in the liver [39]. Because *N. sativa* has various mechanisms of action and hence many biological, pharmacological, and therapeutic effects, MNT was used to evaluate the effects of *N. sativa* when co-administered with CP.

## 2. Materials and Methods

### 2.1. Animals Selection

Twenty healthy six-to-eight-week-old male BALB/c mice were purchased from Bioterio de la Facultad de Medicina y Psicología, Campus Tijuana, Unidad Valle de las Palmas, Universidad Autónoma de Baja California. The mouse model is widely considered the organism of choice for the studies related to human health [40]. The reasons for this selection are numerous, the most important being the relative genetic similarities, the number of protein-coding genes, direct gene alignment between them, and the fact that 99% of human genes are homologous compared to mice [40]. Mice also share many physiologic characteristics with humans and, therefore, makes them the perfect model [40]. Besides the similarities of human and mice species, the reason why the mouse model was selected is that mice do not have a fully developed spleen [32,41,42]. Specifically, BALB/c mice have higher volume density periarterial lymphoid sheaths in the spleen, but lower cytotoxic activity of splenic natural killer (NK) cells compared with other mice strains [43], not allowing micronuclei of circulating erythrocytes to be removed properly. These characteristics make it possible for the visualization of micronuclei in peripheral blood under the microscope, making this model ideal for MNT. In addition to their morphofunctional characteristics, availability and cost-effectiveness were considered at the time of model selection. Mice had an average weight of 22 g and were housed in transparent polycarbonate cages bedded with aspen chips. The animals received standard laboratory pellet food (Purina^®^, Guadalajara, México) and purified water ad libitum. The environment of the animal room was in standard room temperature 20 °C–25 °C and standard room lighting. 

### 2.2. Treatment Regimen

**Figure 1:**
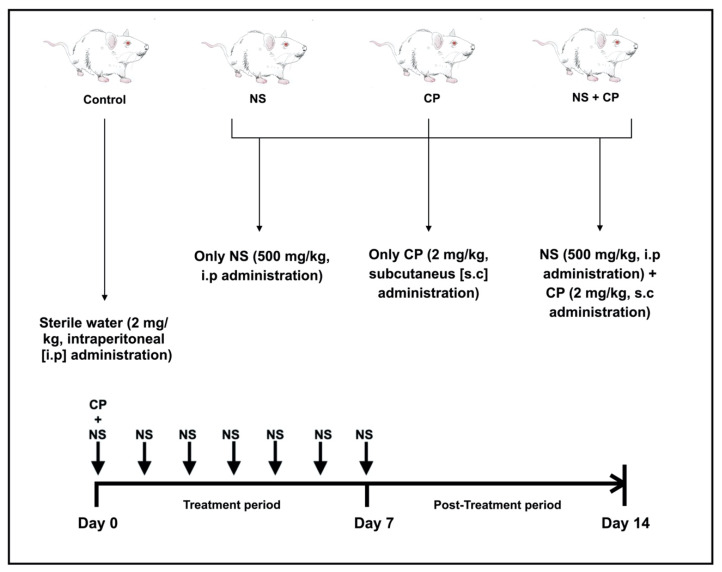
Experimental design. Mice were randomly divided into 4 groups (*n = 5* per group); (1) Control group: single-dose of sterile water (2 mg/kg) was administered i.p on day zero; (2) Ns group: *N. sativa* oil was supplemented (500 mg/kg) i.p daily during the treatment period. (3) CP group: CP was administered in a single-dose (2 mg/kg) s.c diluted with sterile water (CP dose/1ml) on day zero. (4) Ns + CP group: *N. sativa* oil was supplemented (500 mg/kg) i.p daily during the treatment period and CP was administered s.c in a single dose (2 mg/kg) with sterile water (CP dose/1ml) on day zero.

The experiment was conducted for 15 days, of which the first 7 days (treatment period) were for substance administration as well as sample collection. The remaining 7 days (post-treatment period) were for evaluation of the effects of the treatment period through the collection of blood samples. The 15 experimental days include day zero, on which blood samples were drawn prior to administration of any substance. Thereafter, blood samples were collected before administering the respective substance for each group. The BALB/c mice were randomly divided into 4 groups (*n* = 5 per group); (1) Control group: A single dose of sterile water (2 mg/kg) was administered intraperitoneal (i.p) on day zero; (2) Ns group: *N. sativa* oil was administered i.p daily (500 mg/kg) during the treatment period. (3) CP group: CP was administered subcutaneously (s.c) in a single dose (2 mg/kg) diluted with sterile water (CP dose/1mL) on day zero; (4) Ns + CP group: *N. sativa* oil was applied i.p daily (500 mg/kg) during the 7 days of the treatment period and CP was administered s.c in a single dose (2 mg/kg) with sterile water (CP dose/1 mL) on day zero. After the 7th day of the treatment period, blood sample collection continued in all groups until day 14 which is the last day of the experiment (Figure 1).

### 2.3. Compounds

*N. sativa* oil from Seychelles Organics, Inc.^®^ (Park City, UT, USA) was used for this study. It was purchased from Heritage Store, Bowling Green, FA EU, and it is California Certified Organic Farmers (CCOF). The selected dosage (500 mg/kg) of *N. sativa* was based on previously published experiments [44]. The selected dosage (2 mg/kg) of CP from Noveldexis^®^ was based on previously published experiments. [38]

### 2.4. Sample Preparation

One drop of peripheral blood (50 µL) was collected from the tip of the tail of each mouse on day zero of the experiment and daily thereafter for a total of 15 days. Smears were prepared on precleaned labeled microscope slides. The smears were fixed using 80% ethanol for 10 min and allowed to air-dry. Then, the fixed slides were stained with acridine orange (Sigma Aldrich Chemistry lot#MKBD5351V) for 70 s and then placed in phosphate-buffered saline for 5 min. Afterward, slides were air-dried and then stored [17,34,45,46].

### 2.5. Sample Analysis

All slides were labeled before microscopic analysis. Samples were scored manually using a Carl Zeiss^®^, Mod. Axiostar Plus microscope (White Plains, NY, USA) equipped with epi-fluorescence and an oil-immersion objective (100×). The number of PCE in 10,000 total erythrocytes (TE) was determined to assess cytotoxicity. To evaluate acute genotoxicity, we counted the number of micronucleated polychromatic erythrocytes (MNPCE) in 1000 PCE. Finally, to evaluate accumulated genotoxicity, the number of micronucleated erythrocytes (MNE) in 10,000 TE were counted [42,43,44]. 

### 2.6. Statistical Analysis

Results were evaluated using the GraphPad Prism version 8 (San Diego, CA, USA). In the statistical analysis, the data were divided into intragroup and intergroup. Intragroup: The intra-group comparison was made between each treatment group. The comparison was made through repeated measures analysis of variance (ANOVA), followed by a Bonferroni test to correct the significance values of the multiple post hoc pairwise comparisons. More specifically, where the respective baseline value (day zero) was compared with each of the following 14 days of the experiment individually, but not between any other day. The intergroup comparison was made using a one-way ANOVA, followed by Dunnett t-test. More specifically, each of the 15 experimental days within the Control group were individually compared with the respective days of each of the other groups. A *p*-value of <0.05 was considered statistically significant.

### 2.7. Ethical Considerations

The experiment was approved by the institutional animal care and use committee of Universidad Autónoma de Guadalajara (TPI-05-027-1125-19-02) and were performed in accordance with those approved by National (México; Norma Oficial Mexicana NOM-062-ZOO-1999).

## 3. Results

### 3.1. General Characteristics

**Figure 2:**
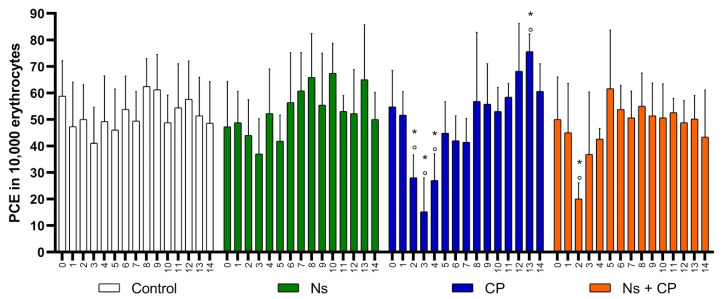
Mean and SD of Polychromatic Erythrocytes in study groups. (PCE-Polychromatic Erythrocytes; Ns-*Nigella Sativa* (500 mg/kg); CP-Cisplatin (2 mg/kg); X axis: Smears every day; °- *p* < 0.05 Intragroup comparison; *- *p* < 0.05 Intergroup comparison).

**Figure 3:**
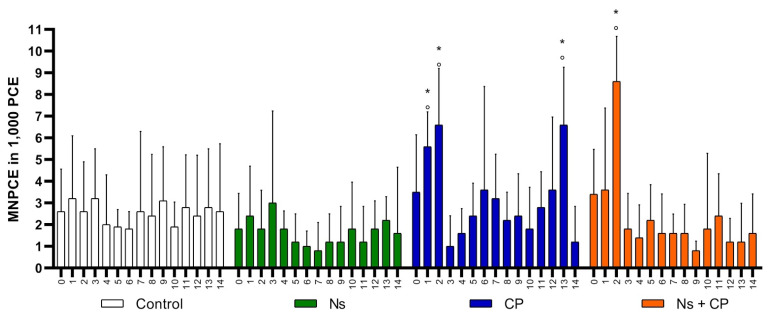
Mean and SD of Micronucleated Polychromatic Erythrocytes in study groups. (MNPCE-Micronucleated Polychromatic Erythrocytes; Ns-*Nigella Sativa* (500 mg/kg); CP-Cisplatin (2 mg/kg); X axis: Smear each day; °- *p* < 0.05 Intragroup comparison; *- *p* < 0.05 Intergroup comparison).

**Figure 4:**
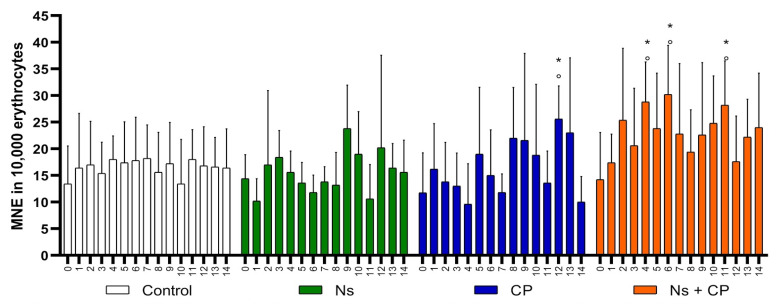
Mean and SD of Micronucleated Erythrocytes in study groups. (MNE-Micronucleated Erythrocytes; Ns-*Nigella Sativa* (500 mg/kg); CP-Cisplatin (2 mg/kg); X axis: Smear each day; °- *p* < 0.05 Intragroup comparison; *- *p* < 0.05 Intergroup comparison).

The spleen plays many physiologic vital roles in the body; one important role to mention is that it acts as a filter to remove damaged hematopoietic cells in peripheral blood [41,42], and this is why it is important to note that variables cannot reach zero. This is because mice do not have a fully developed spleen in comparison to other species, which is why this model is ideal for MNT [32,41,42]. All the variables within the groups started at a baseline with no statistically significant difference between them, meaning that the groups are homogenous, as can be seen in Table 1 during day zero. Average, standard deviation and statistically significant differences of PCE, MNPCE, and MNE are shown in Table 1 and Figure 2, Figure 3 and Figure 4. It is important to note that the control group was considered as a negative control, and the CP group was used as a positive control.

### 3.2. Cytotoxicity Analysis

The ability of a substance to cause cytotoxicity is measured by its capability to decrease cell proliferation. Moreover, in the present study, cytotoxicity is measured by its effect on bone marrow suppression (myelosuppression), which can be interpreted as a reduction of PCE. On the other hand, an increase in the frequency of PCE indicates a myeloproliferative effect. To evaluate the cytotoxicity of the substances administered, we counted the PCE in 10,000 TE. The supplementation of *N. sativa* (500 mg/kg) shows an increased frequency of PCE between days 3 and 10, shown in Figure 2. The increase in the frequency of PCE between these days may indicate a myeloproliferative effect in this organism because, as previously mentioned, an increase in the frequency of PCE would be interpreted as better productivity of the bone marrow.

Analyzing the Control and Ns groups, both showed similar behavior in the frequency of PCE. There were no statistically significant differences (*p* < 0.05) throughout the experiment (Table 1), in comparison with the control group which is not cytotoxic. Moreover, the cytotoxic effect of CP (2 mg/kg) was seen within three statistical differences, between days 1–3, as can be seen in Figure 2, where there is a decrease in the frequency of PCE [47]. This means that there was myelosuppression caused by CP. Thereafter, the organism underwent an adaptative response, which can be defined as a regulation of the frequency of PCE. In this group specifically, the regulation was perceived as an increase in the frequency of PCE since day 4, where the bone marrow uncontrollably allows cell proliferation reaching a maximum point of 75.6 ± 6.7 EPC, on day 13 of the study (Table 1). 

In the Ns + CP group, the cytotoxic effect of CP appears two days after its administration, showing a statistically significant difference of 20.0 ± 6.1 EPC. However, in the supplementation of *N. sativa* for 7 days, bone marrow recovered at a faster rate, and the frequency of EPC was regulated on days 6–14, in comparison to the CP group (Figure 2). The results suggest that the effects of CP cannot be inhibited, but when *N. sativa* is administered as an adjuvant, the bone marrow recovery is faster, and it modulates the cytotoxic effect of CP.

### 3.3. Acute Genotoxicity Analysis

Acute genotoxicity is measured by the appearance of micronuclei in polychromatic erythrocytes, and its prevalence can only be seen under the microscope within the first 24 h of their emergence. An increase in MNPCE means acute genotoxicity, but a decrease indicates otherwise. To evaluate acute genotoxicity of the substances administered, we counted MNPCE in 1,000 PCE. Comparing the Control group with the Ns group, they both showed a similar frequency of MNPCE throughout the study, where there is no statistically significant difference (*p* < 0.05) as shown in Figure 3. Thus, *N. sativa* does not have an acute genotoxic effect in this organism.

The CP group shows an increase in the frequency of MNPCE on days 1 and 2, and, as previously mentioned, this reflects acute genotoxicity, as is shown in Figure 3. Moreover, the CP group showed a statistically significant difference in the frequency of MNPCE of 6.6 ± 2.7 on day 13 as shown in Table 1 and Figure 3. It should be highlighted that, at this time, the cell division rate represented by the frequency of PCE is at its maximum peak. 

The acute genotoxic effect in the CP group was appreciated in the first two days (both day 1 and day 2). On the other hand, in the Ns + CP group, the acute genotoxicity effect was not seen until day 2 (Figure 3). This infers that the genotoxicity effect is delayed with the supplementation of Ns with CP. Thereafter, regulation of the MNPCE was observed throughout the rest of the study. These results suggest that *N. sativa* has a genoprotective effect against the acute damage of CP. It accomplishes this by regulating and then maintaining a stable frequency of MNPCE throughout the experiment.

### 3.4. Accumulated Genotoxicity Analysis

Accumulated genotoxicity is measured through the frequency of MNE; its effect is directly proportional to the frequency of MNE. To evaluate the accumulated genotoxicity of the substances administered, we counted the frequency of MNE in 10,000 TE. Comparing the Control group with the Ns group, they both showed a similar frequency of MNE throughout the study, where there is no statistically significant difference (*p* < 0.05) as shown in Figure 4. Thus, *N. sativa* does not have an acute genotoxic effect in this organism.

The CP group showed a statistically significant difference in the frequency of MNE of 25.6 ± 6.2 MNE on day 12 as is shown in Table 1 and Figure 4. In group Ns + CP, the frequency of MNE increased, as seen in three statistically significant different points on days 4, 6, and 11 (Figure 4). Moreover, the presence of MNE is higher when there is an increased rate of cell division, where both statistical points can be observed on day 12 in the CP group (Figure 4). These results suggest that the combination of *N. sativa* with CP is more effective at enhancing the anti-cytotoxic effects rather than the overall genoprotective effect in this organism. This can be seen by the formation of MNE from the increased rate of cell division, but this does not imply that *N. sativa* has a genotoxic effect. 

## 4. Discussion

*N. sativa* is among the most used medicinal herbs throughout the world. Its use dates back to ancient times while its scientific study has gained popularity in the last few decades. It is widely known that medicinal plants not only have therapeutic effects but may also possess toxic effects associated with their phytochemical compounds; for this reason, they must be subject to toxicological tests, including those for cytotoxicity and genotoxicity [48,49]. More specifically, the numerous studies done on *N. sativa* have reported its therapeutic pharmacological effects, which have been established to be diuretic, analgesic, anthelmintic, bronchodilator, spasmolytic, gastroprotective, hepatoprotective, as well as anticancer, antioxidant, antihypertensive, antidiabetic, and immunomodulation [1,2,3,4,6,9,10,11,12,13,14,50,51,52,53,54]. However, the anti-cytotoxic and anti-genotoxic properties have never been evaluated and, therefore, in the present study, these properties, for the first time, will be evaluated through MNT of BALB/c mice peripheral blood. 

In this study, the result of PCE, MNPCE, and MNE frequencies showed similar behavior, throughout the experiment when *N. sativa* was administered, meaning that it is a non-toxic substance. Other studies also report no evidence of *N. sativa* toxicity in different cell lines [55,56]. As reported, it is impossible for the biomarkers used to show a fixed frequency throughout the study. It should be taken into consideration that we are studying a living model and therefore physiological as well as external factors contributed to fluctuations within the biomarkers frequency results but did not have significant statistical differences.

The results in the evaluation of the anti-cytotoxic effect of substances suggest that the effects of CP cannot be inhibited, but when *N. sativa* is administered as an adjuvant, not only is the bone marrow recovery faster but also modulates the cytotoxic effect of CP which is reflected in the frequency of EPC. In other studies, the myelosuppression effect was also inhibited by *N. sativa*, proving that it has anti-cytotoxic properties [54]. Our findings also agree with previously published studies [38,39] which state that *N. sativa* components have an effective cell proliferation and regulation of the adaptive response of the organism. It also prevents the weakening of the immune system and protects healthy cells from oxidative damage, preventing toxic side-effects [39,55], and proving great stability in cell and bone marrow recovery.

Moreover, these results suggest that *N. sativa* has a genoprotective effect against the toxic side effects of CP. The genotoxic effects of CP increased since the beginning of the treatment period, which resulted from the decreased rate and inefficiency of healthy cell division. A delayed increase in the formation and the ability to regulate MNPCE in MNT was observed after the supplementation of *N. sativa* oil, suggesting that *N. sativa* has a genoprotective effect against the toxic effects of CP. It has been reported in other investigations that *N. sativa* has genoprotective properties [57,58]. Therefore we used the MNT to evaluate the effect of *N. sativa* when used as an adjuvant with CP.

Many research studies explain that the combination of the various mechanisms of action of *N. sativa* gives its many therapeutic, biological, and pharmacological effects, allowing alkylating agents like CP to act on the malignant cells but not on healthy cells. Moreover, the cytoprotective and genoprotective activity of *N. sativa* is corroborated by the MNT. We suggest that *N. sativa* can function as an adjuvant to drugs that cause generalized toxicity in the organism since it can aid in their mechanisms of action [39] but also prevent them from acting on healthy cells, as our result suggests that *N. sativa* effectively proliferates cell division and regulates the adaptive response of the organism. Based on previous studies and the results obtained from our study, further research is needed to evaluate the behavior of *N. sativa* in different case scenarios. 

## 5. Conclusions

In conclusion, it is evident that *N. sativa* has a wide spectrum of beneficial properties, which is why it has been considered a miracle herb; and it has been targeted in the research areas of new drug development for potential use as an adjuvant. Our results indicate that *N. sativa* does not induce cytotoxic and genotoxic effects on BALB/c mice. Therefore, by the evaluation of the frequency of PCE, MNPCE, and MNE biomarkers through the MNT, we conclude that *N. sativa* has cytoprotective, genoprotective effects and modulates cell proliferation in BALB/c mice.

## Figures and Tables

**Table 1:** PCE, MNPCE, MNE, and frequencies from mice study groups.

Day	Control			Ns			CP			Ns + CP		
	PCE	MNPCE	MNE	PCE	MNPCE	*MNE*	PCE	MNPCE	MNE	PCE	MNPCE	MNE
0	58.8 ± 13.4	2.6 ± 2.0	13.4 ± 7.2	47.2 ± 17.1	1.8 ± 1.6	14.4 ± 4.5	54.8 ± 13.7	3.5 ± 2.6	11.8 ± 7.5	50.0 ± 16.0	3.4 ± 2.1	14.2 ± 8.9
				WS *	WS *	WS *	WS *	WS *	WS *	WS *	WS *	WS *
1	47.3 ± 16.8 WS °	3.2 ± 2.9 WS °	16.4 ± 10.2 WS °	48.8 ± 11.8	2.4 ± 2.3	10.2 ± 4.2	51.6 ± 8.9	5.6 ± 1.6	16.2 ± 8.5	45.0 ± 18.6	3.6 ± 3.8	17.4 ± 5.4
				WS *	WS *	WS *	WS *	*p <* 0.04 *	WS *	WS *	WS *	WS *
				WS °	WS °	WS °	WS °	*p <* 0.01 °	WS °	WS °	WS °	WS °
2	50.0 ± 13.1 WS °	2.6 ± 2.3 WS °	17.0 ± 8.2 WS °	44.0 ± 13.5	1.8 ± 1.8	17.0 ± 13.9	28.0 ± 8.7	6.6 ± 2.6	13.8 ± 7.4	20.0 ± 6.1	8.6 ± 2.1	25.4 ± 13.5
				WS *	WS *	WS *	*p <* 0.001 *	*p <* 0.01 *	WS *	*p <* 0.002 *	*p <* 0.0001 *	WS *
				WS °	WS °	WS °	*p <* 0.002 °	*p <* 0.001 °	WS °	*p <* 0.001 °	*p <* 0.0001 °	WS °
3	41.0 ± 13.7 WS °	3.2 ± 2.3 WS °	15.4 ± 5.9 WS °	37.0 ± 13.3	3.0 ± 4.2	18.4 ± 5.0	15.2 ± 12.7	1.0 ± 1.4	13.0 ± 6.2	36.8 ± 23.5	1.8 ± 1.6	20.6 ± 10.8
				WS *	WS *	WS *	*p <* 0.0001 *	WS *	WS *	WS *	WS *	WS *
				WS °	WS °	WS °	*p <* 0.0001 °	WS °	WS °	WS °	WS °	WS °
4	49.2 ± 17.2 WS °	2.0 ± 2.3 WS °	18.0 ± 4.4 WS °	52.2 ± 16.8	1.8 ± 0.8	15.6 ± 4.0	27.0 ± 10.1	1.6 ± 1.1	9.6 ± 7.6	42.6 ± 4.0	1.4 ± 1.5	28.8 ± 7.5
				WS *	WS *	WS *	*p <* 0.001 *	WS *	WS *	WS *	WS *	*p <* 0.03 *
				WS °	WS °	WS °	*p <* 0.002 °	WS °	WS °	WS °	WS °	*p <* 0.01 °
5	46.0 ± 15.5 WS °	1.9 ± 0.8 WS °	17.4 ± 7.7 WS °	41.8 ± 9.9	1.2 ± 1.3	13.6 ± 3.8	44.8 ± 11.9	2.4 ± 1.5	19.0 ± 12.6	61.6 ± 22.1	2.2 ± 1.6	23.8 ± 10.4
				WS *	WS *	WS *	WS *	WS *	WS *	WS *	WS *	WS *
				WS °	WS °	WS °	WS °	WS °	WS °	WS °	WS °	WS °
6	53.8 ± 12.6 WS °	1.8 ± 0.8 WS °	17.8 ± 8.1 WS °	56.4 ± 18.8	1.0 ± 0.7	11.8 ± 3.3	42.0 ± 9.4	3.6 ± 4.8	15.0 ± 8.6	53.8 ± 9.0	1.6 ± 1.8	30.2 ± 9.2
				WS *	WS *	WS *	WS *	WS *	WS *	WS *	WS *	*p <* 0.01 *
				WS °	WS °	WS °	WS °	WS °	WS °	WS °	WS °	*p <* 0.01 °
7	49.4 ± 11.1 WS °	2.6 ± 3.7 WS °	18.2 ± 6.3 WS °	60.8 ± 14.4	0.8 ± 1.3	13.8 ± 2.9	41.4 ± 9.0	3.2 ± 2.0	11.8 ± 3.5	50.6 ± 10.1	1.6 ± 0.9	22.8 ± 13.2
				WS *	WS *	WS *	WS *	WS *	WS *	WS *	WS *	WS *
				WS °	WS °	WS °	WS °	WS °	WS °	WS °	WS °	WS °
8	62.4 ± 10.5 WS °	2.4 ± 2.8 WS °	15.6 ± 7.5 WS °	65.8 ± 16.6	1.2 ± 1.3	13.2 ± 6.1	56.8 ± 26.1	2.2 ± 1.3	22.0 ± 9.5	55.0 ± 12.5	1.6 ± 1.3	19.4 ± 7.9
				WS *	WS *	WS *	WS *	WS *	WS *	WS *	WS *	WS *
				WS °	WS °	WS °	WS °	WS °	WS °	WS °	WS °	WS °
9	61.2 ± 13.3 WS °	3.1 ± 2.5 WS °	17.2 ± 7.8 WS °	55.4 ± 19.6	1.2 ± 1.6	23.8 ± 8.2	55.8 ± 15.2	2.4 ± 1.9	21.6 ± 16.3	51.4 ± 12.3	0.8 ± 0.4	22.6 ± 13.6
				WS *	WS *	WS *	WS *	WS *	WS *	WS *	WS *	WS *
				WS °	WS °	WS °	WS °	WS °	WS °	WS °	WS °	WS °
10	48.8 ± 10.4 WS °	1.9 ± 1.1 WS °	13.4 ± 8.4 WS °	67.4 ± 11.3	1.8 ± 2.2	19.0 ± 8.0	53.0 ± 9.1	1.8 ± 1.9	18.8 ± 13.3	50.6 ± 12.8	1.8 ± 3.5	24.8 ± 8.9
				WS *	WS *	WS *	WS *	WS *	WS *	WS *	WS *	WS *
				WS °	WS °	WS °	WS °	WS °	WS °	WS °	WS °	WS °
11	54.4 ± 16.6 WS °	2.8 ± 2.4 WS °	18.0 ± 5.6 WS °	53.0 ± 6.0	1.2 ± 1.6	10.6 ± 6.5	58.4 ± 5.1	2.8 ± 1.6	13.6 ± 6.0	52.6 ± 5.4	2.4 ± 1.9	28.2 ± 8.3
				WS *	WS *	WS *	WS *	WS *	WS *	WS *	WS *	*p <* 0.02 *
				WS °	WS °	WS °	WS °	WS °	WS °	WS °	WS °	*p <* 0.03 °
12	57.6 ± 14.4 WS °	2.4 ± 2.8 WS °	16.8 ± 7.4 WS °	52.2 ± 16.6	1.8 ± 1.3	20.2 ± 17.4	68.2 ± 18.0	3.6 ± 3.4	25.6 ± 6.2	48.8 ± 8.4	1.2 ± 1.1	17.6 ± 8.6
				WS *	WS *	WS *	WS *	WS *	*p <* 0.03 *	WS *	WS *	WS *
				WS °	WS °	WS °	WS °	WS °	*p <* 0.01 °	WS °	WS °	WS °
13	51.4 ± 14.5 WS °	2.8 ± 2.7 WS °	16.6 ± 5.5 WS °	65.0 ± 20.7	2.2 ± 1.1	16.4 ± 4.6	75.6 ± 6.7	6.6 ± 2.7	23.0 ± 14.1	50.2 ± 8.9	1.2 ± 1.8	22.2 ± 7.1
				WS *	WS *	WS *	*p <* 0.0001 *	*p <* 0.001 *	WS *	WS *	WS *	WS *
				WS °	WS °	WS °	*p <* 0.0001 °	*p <* 0.002 °	WS °	WS °	WS °	WS °
14	48.6 ± 15.7 WS °	2.6 ± 3.1 WS °	16.4 ± 7.4 WS °	50.0 ± 10.2	1.6 ± 3.0	15.6 ± 6.0	60.6 ± 10.4	1.2 ± 1.6	10.0 ± 4.8	43.4 ± 17.7	1.6 ± 1.8	24.0 ± 10.2
				WS *	WS *	WS *	WS *	WS *	WS *	WS *	WS *	WS *
				WS °	WS °	WS °	WS °	WS °	WS °	WS °	WS °	WS °

Table 1. Data are expressed as mean ± standard deviation. Sample size (*n*). groups: (1) Control (sterile water) (2 mg/kg); (2) *Nigella sativa* (Ns) (500 mg/kg); (3) Cisplatin (CP) (2 mg/kg); (4) Ns + CP (respective dosage). Polychromatic erythrocytes (PCE) were counted over 10,000 total erythrocytes (TE); Micronucleated polychromatic erythrocytes (MNPCE) were evaluated over 1000 PCE; Micronucleated erythrocytes (MNE) were evaluated over 10,000 TE; WS (Without significance) °:Intra-group, comparisons were made each treatment group and their respective control group (day 0); *: Inter-group comparisons were made between treatment groups and their respective control group (0–14 days).
